# Invasions by Eurasian Avian Influenza Virus H6 Genes and Replacement of Its North American Clade

**DOI:** 10.3201/eid1507.090245

**Published:** 2009-07

**Authors:** Heinrich zu Dohna, Jinling Li, Carol J. Cardona, Joy Miller, Tim E. Carpenter

**Affiliations:** University of California School of Veterinary Medicine, Davis, California, USA (H. zu Dohna, J. Li, C.J. Cardona, T.E. Carpenter); National Center for Medical Intelligence, Fort Detrick, Maryland, USA (J. Miller); 1 These authors contributed equally to this article.

**Keywords:** H6, phylogeny, Eurasia, North America, hemisphere, influenza, avian influenza, migration, viruses, research

## Abstract

The spread of highly pathogenic avian influenza virus (AIV) (H5N1) underlines the potential for global AIV movement through birds. The phylogenies of AIV genes from avian hosts usually separate into Eurasian and North American clades, reflecting limited bird migration between the hemispheres. However, mounting evidence that some H6 sequences from North America cluster with Eurasian subtype H6 sequences calls the strict hemispheric divide into question. We conducted a comprehensive phylogenetic analysis of the extent and timing of cross-hemisphere movements by the H6 gene. Results suggested that Eurasian H6 subtype has invaded North America several times, with the first invasions occurring 10 years before the first detection of invading isolates. The members of the North American clade decreased from 100% in the 1980s to 20% in the 2000s among H6 isolates from North America. Unraveling the reasons for this large-scale gene movement between hemispheres might identify drivers of global AIV circulation.

## Abstract 2

This study showed frequent cross-hemisphere virus movement, which can affect the risk posed to poultry and humans.

The transboundary expansion of highly pathogenic avian influenza virus (AIV) (H5N1) in 2005 increased the interest in global AIV spread by wild birds (*1*,*2*). Although the degree to which wild birds contribute to the large-scale spread of highly pathogenic subtype H5N1 influenza viruses has been much debated (*3*–*5*), consensus exists that natural barriers limit the movement of the virus between the Eastern and Western Hemispheres (*2*,*6*). To date, the spread of highly pathogenic AIV subtype H5N1 has been confined to Eurasia and Africa. Understanding the potential for genetic interchange of AIV between hemispheres is critical to limiting spread and mitigating the effects of AIV on human and animal health (*1*). Phylogenetic trees of most AIV gene segments and subtypes demonstrate a clear separation between North American and Eurasian clades, reflecting limited virus movement through bird migration between the hemispheres (*7*). However, some subtype H6 AIV strains isolated in North America were recently found to cluster in the Eurasian clade (*8*–*10*), which calls the belief in a strict hemispheric divide into question.

Subtype H6 (hereafter called H6) AIV infection occurs frequently in wild and domestic birds in Asia, America, and Africa (*11*–*16*). Although H6 is a subtype with low pathogenicity, outbreaks of H6 AIV in domestic birds have had a serious impact on the poultry industry in California (*17*). In addition, recent reports indicate a subtype H6N1 virus, A/teal/HK/W312/97, shared a common source of 6 of 8 gene fragments with a subtype H5N1 virus, A/Hong Kong/156/97 (H5N1) (*18*–*20*); the latter caused an outbreak among chickens, with sporadic human cases and deaths in Hong Kong during 1997 (*21*–*23*).

Given the importance of cross-hemisphere AIV movement for the United States, evidence for cross-hemisphere H6 AIV movement deserves closer examination. In this study, we investigated whether previous indications of cross-hemisphere movement of H6 AIV were part of a larger viral movement pattern. We constructed a phylogenetic tree using all currently available full-length H6 sequences and analyzed the spatial and temporal distribution of all sequences that showed a mismatch between genetic and geographic proximity to other sequences.

## Methods

### Nucleotide Sequence Data

Nucleotide sequences and information on host, sampling date, and location were obtained for all H6 nucleotide sequences from the National Center for Biotechnology Information Influenza Sequence Database and BioHealthBase (*24*,*25*) by using the following keywords: influenza A, H6, and avian host. All H6 sequences above the length of 1,650 nt were retained, and duplicate sequences of the same isolates were removed, leading to a total of 291 H6 sequences. These sequences were aligned with AlignX (Vector NTI version X; Invitrogen, Carlsbad, CA, USA), and a maximum-likelihood phylogenetic tree was built using the *R* package ape (*26*). The tree was based on a substitution model that was first proposed by Felsenstein (*27*) and is now standard for maximum likelihood estimation in most phylogenetic software packages (e.g., see p. 104 in *26*). This substitution model enables different rates for transitions and transversions, unequal base frequencies, and different substitution rates for wobble positions (*26*). The oldest H6 isolate was used as the root. The support of each bipartition was determined from 100-bootstrap samples. Other substitution models and neighbor-joining methods to construct phylogenetic trees produced very similar trees (H. zu Dohna, unpub. data).

### Cross-Hemisphere Movement

Within the phylogenetic tree for the H6 gene, the 2 largest clades were determined and labeled as either North American or Eurasian, depending on where most isolates in each clade were found. All exceptions to this pattern, i.e., all isolates that clustered in a clade from a hemisphere that differed from their sampling location, were analyzed further. Following the analysis of Krauss et al. (*6*), these isolates were considered to have invaded a new hemisphere and will be referred to as invading isolates. The hemisphere to which the invading isolates belong genetically, as determined by the clade in which they cluster, will be referred to as the old hemisphere and the hemisphere from which they were isolated as the new hemisphere. All invading isolates were grouped into monophyletic invading subclades to estimate the times of invasion events.

**Figure 1:**
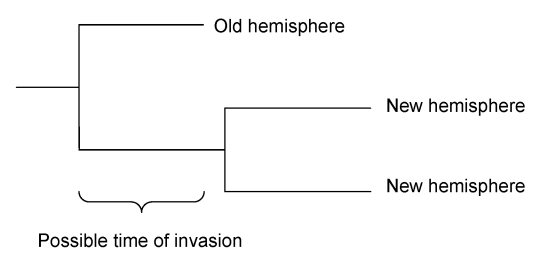
Possible period of invasion from the old hemisphere to the new hemisphere shown in a schematic tree with 2 isolates from the new and 1 from the old hemisphere.

For each invading subclade, 2 time points that bracket the most parsimonious invasion time were estimated. An invasion event that gave rise to a subclade must have happened after the last branching that led to descendents in the old hemisphere and before the time of the most recent common ancestor (TMRCA) for all descendents in the new hemisphere (Figure 1). These 2 time points were approximated for each subclade by estimating the TMRCAs for 2 sets of sequences, either including or excluding the most closely related sequence from the old hemisphere (Figure 1). This method assumes that invading isolates that are more closely related to each other than they are to other isolates from the old hemisphere descended from the same invasion event. This assumption is most parsimonious since there should be a low probability that the descendents of an invading virus reach a high enough density to be detected in bird samples several years later.

TMRCAs were obtained by fitting a coalescent process to the gene sequence data, by using the Bayesian Monte Carlo Markov Chain package BEAST 1.4 (*28*). A general time-reversible substitution model with variable population size (*29*) was fitted to nucleotide data. To maximize conformity with model assumptions, only the wobble positions of conserved amino acids with 4-fold degenerate codons were chosen. The program returns sampled posterior distributions of each TMRCA estimate. The Markov chain was run for 5 million steps and parameters were sampled every 1,000th step.

The posterior distribution *f*(*t*) of the time of invasion *t* was determined from all sampled pairs of the time of the earlier node and later node, *t_ei_* and *t_li_*, respectively:
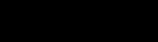
,where the sum was taken over all pairs *i*, *I* denotes an indicator variable that equals 1 if *t_ei_* < *t* < *t_li_* and 0 otherwise, and *c* is a normalizing constant ensuring that *f*(*t*) sums to 1 when summed over all times *t*. Additional analysis showed that the results were very similar when all nucleotide positions were included or when the HKY 85 substitution model (*30*) was chosen. However, the method described above produced the tightest posterior distributions for TMRCAs.

To determine whether invasion events had an effect on clade composition, the temporal change of the probability that an isolate was sampled from the North American clade was fitted by logistic regression using the statistical software *R* (*31*). The data were grouped by hemisphere and host type (wild versus domestic birds) and analyzed for each group separately since wild and domestic birds were sampled differently. The wild bird analysis was based on individual isolates and the domestic bird analysis on the number of introductions into the domestic poultry species. Because the analysis relied on publicly available data, only introductions into domestic poultry that led to a sequence submission were analyzed. H6 sequences from domestic bird isolates in different years were counted as separate introductions. For all neuraminidase (NA) sequences that were part of a virus isolate whose H6 gene was classified as invader, the clade membership of the NA gene was determined as well. In addition, the proportion of H6 among all hemagglutinin sequences (any length, but only 1 sequence per isolate) from long-term wild bird survey sites in Alberta, Canada, and Ohio and Delaware, USA (*25*), was calculated per decade. These sites were chosen because AIV isolates were sampled from these sites following a standardized sampling regimen for more than a decade (*32*).

## Results

**Figure 2:**
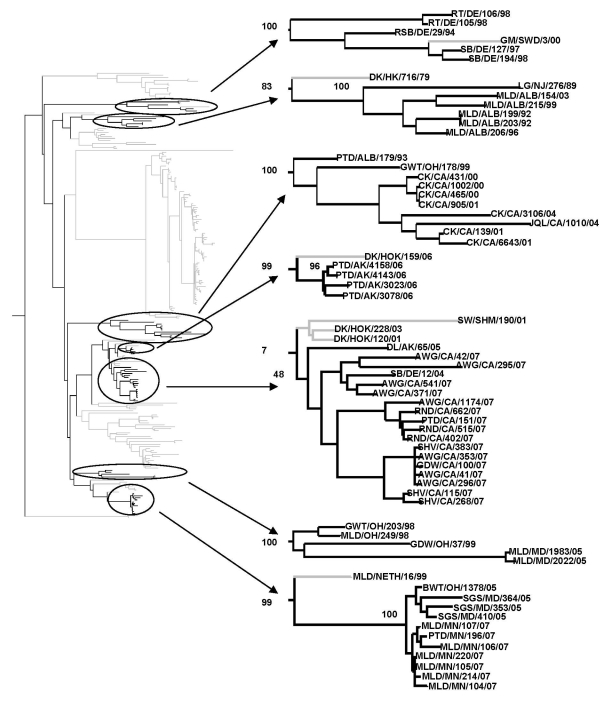
Eurasian clade of the phylogenetic tree for all full-length H6 sequences of avian influenza virus (excluding multiple sequences of the same isolate). Black branches indicate isolates from North America, and gray branches indicate isolates from Eurasia. The 7 subclades that invaded North America and their closest Eurasian related clade and bootstrap values are shown on the right. Abbreviations for strain names are listed in the Technical Appendix Table.

The phylogenetic tree of all 291 full-length H6 gene sequences shows a clear division between the Eurasian and North American clades (Technical Appendix Figure 1). Within the Eurasian clade there were 7 monophyletic subclades composed of strains isolated from North America (Figure 2). Six of these 7 subclades had bootstrap support above 90%. Five of these 7 subclades were closely related to isolates in Eurasia. Of these 5 subclades, 3 were most closely related to isolates in East Asia, and 2 were most closely related to clades in Northern Europe. All North American isolates of the 2 subclades most closely related to clades in Europe were found on the East Coast or in the Midwest region of the United States. Most of the isolates from the subclades closely related to isolates in Asia were found in Alberta, Canada, and the West Coast of the United States. The exceptions were 1 isolate from New Jersey and 1 from Delaware, which clustered in subclades with close relatives in Asia (Figure 2).

**Figure 3:**
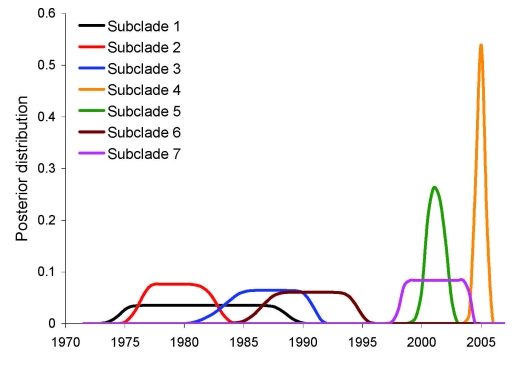
Posterior distribution of estimated invasion times of the 7 subclades of Eurasian avian influenza virus subtype H6 that invaded North America. Numbering of subclades corresponds to the order within the phylogeny of Figure 2 (top = 1, bottom = 7).

**Figure 4:**
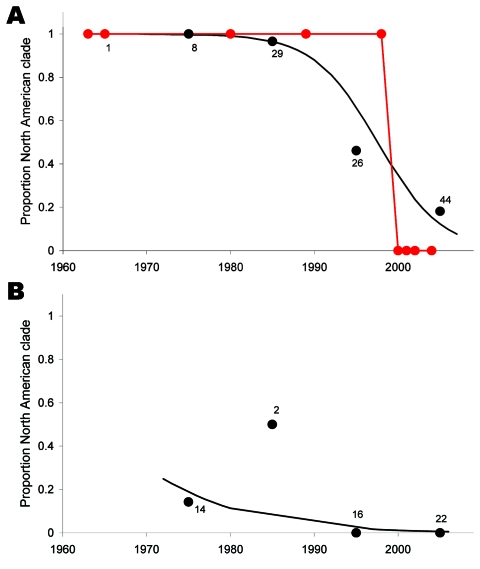
Proportion of viruses from the North American clade of avian influenza virus among wild bird isolates (blue) or poultry outbreaks (pink) in North America (A) or Eurasia (B). All poultry outbreaks in Eurasia were caused by viruses from the Eurasian clade (results not shown). Wild bird isolates are grouped by decade. Black circles show proportions of all isolates per decade (number of isolates per decade shown next to circles), black lines show fitted logistic regressions, and red circles show individual poultry outbreaks.

The estimated entry times of these 7 subclades overlapped in part, with the earliest times starting in the late 1970s and the latest around 2005 (Figure 3). The proportion of North American clade members among isolates from North American wild birds decreased from 100% in the 1980s to 20% in the 2000s (Figure 4; p<0.0001, logistic regression, residual deviance = 75, df = 106). Similarly, all H6 introductions into poultry species in North America with sequences submitted to the public databases were caused by viruses from the North American clade before 1998 and by viruses from the Eurasian clade after 1999 (Figure 4). All 38 H6 sequences isolated in North America after 2002 belong to the Eurasian clade. The change in clade composition among North American viruses is visible across the entire North American continent (Technical Appendix Figures 2, 3). One subclade invaded Australia from North America but did not result in a significant impact on the clade composition over time in Eurasia (Figure 4, panel B). The increase of Eurasian H6 among North American H6 isolates did not coincide with an overall increase of H6 prevalence among AIV isolates reported in wild birds in North America (results not shown). All NA genes that were combined with invading H6 genes belonged to North American clades (Technical Appendix Figures 4–7).

## Discussion

A comprehensive phylogenetic analysis of all currently available full-length H6 gene sequences showed a major change in clade composition among isolates from North America. Seven subclades within the Eurasian clade were composed of isolates from North America. Isolates from these subclades started to appear in North America in the 1990s and became highly dominant among North American isolates by the early 2000s. This pattern provides strong evidence for multiple invasions of North America by Eurasian H6 strains that led to the replacement of the North American H6 clade. Previous studies that addressed cross-hemisphere movement detected evidence for infrequent cross-hemisphere movement (*6*,*7*), but did not show the change of clade composition over time.

The closest native Eurasian relatives of the invading subclades indicated movement of H6 to North America from Northern Europe and East Asia. In addition, some evidence indicates possible movement back from North America to Eurasia (e.g., subclades 6 and 7 as shown in Figure 2 could have resulted from 1 movement from Europe into North America and a second movement from North America back to Europe). Although the phylogenetic analysis cannot assess the actual number of invasion events, it nevertheless indicates that several invasion events occurred. A main limitation of the data is that the sampling system was not consistent across time, space, and host species. For example, no H6 samples from the West Coast of North America are available before 2000; therefore, some of the decline in the proportion of the North American clade among H6 viruses could be attributed to an increase of samples from the West Coast, which contained only members of the Eurasian clade. However, even when isolates are grouped by region, the decline of the North American clade can still be observed (Technical Appendix Figure 2).

Overall, the patterns suggest frequent movement between the hemispheres in the 1990s and 2000s. The deep division between the Eurasian and North American clades would not be expected if this frequent cross-hemisphere movement had occurred in the distant past. We therefore propose that cross-hemisphere virus movement increased in the last few decades. A formal test of this hypothesis would require estimating migration rates over time from phylogenetic data (*33*–*35*), while taking the limitations of the data into account, which is beyond the scope of the present paper. Comparing estimated time-varying virus migration rates with data on bird trade and wild bird migration might shed light on the mechanisms of cross-hemisphere movement.

That the sequences of the invading viruses did not cluster in a single monophyletic group suggests that the invasion events were not triggered by a single genetic change in the H6 gene. The pattern observed in the H6 gene could have been caused by the H6 gene spreading along with the expansion of another gene segment that reassorts rarely with the H6 gene. The analysis of the NA genes shows that no concurrent movement of NA genes occurred. However, we have not analyzed the internal genes. For a more complete understanding of AIV movement, the analysis developed here must be applied to all subtypes and gene segments.

Whether similar invasion events occurred for other gene segments or subtypes is unknown. Another study found that among AIVs, viruses of the H6 subtype have wider host ranges than other AIV subtypes (*13*). This could make H6 virus more prone to spread between and within hemispheres than other AIV subtypes.

Our study has shown that cross-hemisphere AIV movement can lead to a dramatic change in strain composition within a decade that affects an entire continent. We do not know whether expansion of the Eurasian H6 led to a change in risk for the poultry industry since H6 poultry isolates may not be well represented in public gene sequence databases. For example, H6 outbreaks have been documented in poultry operations in Minnesota in 12 different years from 1978 to 2005 (*36*), yet gene sequences of only 1 virus isolate from poultry outbreaks in Minnesota are available in GenBank. Although it is currently not possible to assess the risk posed to poultry by invading H6 AIV, the change in risk for the domestic poultry flocks clearly would be substantial if a similar invasion did occur for the highly pathogenic AIVs that are currently restricted to Eurasia.

This study corroborates a previous study (*6*) that found no evidence of cross-hemisphere invasions by entire virus genomes but detected only invasions of single gene segments that reassorted with other segments found in the new hemisphere. Thus, invasions can lead to new combinations of host range determining factors on different gene segments that were geographically separated before an invasion event. In general, invasions of the scope and speed as the instance documented here have the potential to strongly affect the risk posed by AIV to poultry and humans.

## Supplementary Material

Technical Appendix Invasions by Eurasian Avian Influenza Virus H6 Genes and Replacement of Its North American Clade

## References

[1] Kilpatrick AM, Chmura AA, Gibbons DW, Fleischer RC, Marra PP, Daszak P. Predicting the global spread of H5N1 avian influenza. Proc Natl Acad Sci U S A. 2006;103:19368–73. 10.1073/pnas.0609227103 17158217 PMC1748232

[2] Olsen B, Munster VJ, Wallensten A, Waldenstrom J, Osterhaus AD, Fouchier RA. Global patterns of influenza A virus in wild birds. Science. 2006;312:384–8. 10.1126/science.1122438 16627734

[3] Feare CJ, Yasue M. Asymptomatic infection with highly pathogenic avian influenza H5N1 in wild birds: how sound is the evidence? Virol J. 2006;3:96. 10.1186/1743-422X-3-96 17112378 PMC1661592

[4] Sturm-Ramirez KM, Hulse-Post DJ, Govorkova EA, Humberd J, Seiler P, Puthavathana P, Are ducks contributing to the endemicity of highly pathogenic H5N1 influenza virus in Asia? J Virol. 2005;79:11269–79. 10.1128/JVI.79.17.11269-11279.2005 16103179 PMC1193583

[5] Weber TP, Stilianakis NI. Ecologic immunology of avian influenza (H5N1) in migratory birds. Emerg Infect Dis. 2007;13:1139–43. 17953082 10.3201/eid1308.070319 PMC2828095

[6] Krauss S, Obert CA, Franks J, Walker D, Jones K, Seiler P, Influenza in migratory birds and evidence of limited intercontinental virus exchange. PLoS Pathog. 2007;3:e167. 10.1371/journal.ppat.0030167 17997603 PMC2065878

[7] Dugan VG, Chen R, Spiro DJ, Sengamalay N, Zaborsky J, Ghedin E, The evolutionary genetics and emergence of avian influenza viruses in wild birds. PLoS Pathog. 2008;4:e1000076. 10.1371/journal.ppat.1000076 18516303 PMC2387073

[8] Chen Z, Santos C, Aspelund A, Gillim-Ross L, Jin H, Kemble G, Evaluation of live attenuated influenza A virus H6 vaccines in mice and ferrets. J Virol. 2009;83:65–72. 10.1128/JVI.01775-08 18945773 PMC2612304

[9] Wahlgren J, Waldenstrom J, Sahlin S, Haemig PD, Fouchier RA, Osterhaus AD, Gene segment reassortment between American and Asian lineages of avian influenza virus from waterfowl in the Beringia area. Vector Borne Zoonotic Dis. 2008;8:783–90. 10.1089/vbz.2007.0274 18637721

[10] Webby RJ, Woolcock PR, Krauss SL, Walker DB, Chin PS, Shortridge KF, Multiple genotypes of nonpathogenic H6N2 influenza viruses isolated from chickens in California. Avian Dis. 2003;47(Suppl):905–10. 10.1637/0005-2086-47.s3.905 14575084

[11] Hanson BA, Stallknecht DE, Swayne DE, Lewis LA, Senne DA. Avian influenza viruses in Minnesota ducks during 1998–2000. Avian Dis. 2003;47(Suppl):867–71. 10.1637/0005-2086-47.s3.867 14575079

[12] Wallensten A, Munster VJ, Latorre-Margalef N, Brytting M, Elmberg J, Fouchier RA, Surveillance of influenza A virus in migratory waterfowl in northern Europe. Emerg Infect Dis. 2007;13:404–11. 17552093 10.3201/eid1303.061130 PMC2725893

[13] Munster VJ, Baas C, Lexmond P, Waldenstrom J, Wallensten A, Fransson T, Spatial, temporal, and species variation in prevalence of influenza A viruses in wild migratory birds. PLoS Pathog. 2007;3:e61. 10.1371/journal.ppat.0030061 17500589 PMC1876497

[14] Kinde H, Read DH, Daft BM, Hammarlund M, Moore J, Uzal F, The occurrence of avian influenza A subtype H6N2 in commercial layer flocks in southern California (2000–02): clinicopathologic findings. Avian Dis. 2003;47(Suppl):1214–8. 10.1637/0005-2086-47.s3.1214 14575145

[15] Panigrahy B, Senne DA, Pedersen JC. Avian influenza virus subtypes inside and outside the live bird markets, 1993–2000: a spatial and temporal relationship. Avian Dis. 2002;46:298–307. 10.1637/0005-2086(2002)046[0298:AIVSIA]2.0.CO;2 12061638

[16] Woolcock PR, Suarez DL, Kuney D. Low-pathogenicity avian influenza virus (H6N2) in chickens in California, 2000–02. Avian Dis. 2003;47(Suppl):872–81. 10.1637/0005-2086-47.s3.872 14575080

[17] Cardona C. Low pathogenicity avian influenza outbreaks in commercial poultry in California. In: Knobler SL, Mack A, Mahmoud A, Lemon SM, editors. The threat of pandemic influenza: are we ready? Washington: National Academies Press; 2005. p. 243–53. 20669448

[18] Cheung CL, Vijaykrishna D, Smith GJ, Fan XH, Zhang JX, Bahl J, Establishment of influenza A virus (H6N1) in minor poultry species in southern China. J Virol. 2007;81:10402–12. 10.1128/JVI.01157-07 17652385 PMC2045442

[19] Chin PS, Hoffmann E, Webby R, Webster RG, Guan Y, Peiris M, Molecular evolution of H6 influenza viruses from poultry in southeastern China: prevalence of H6N1 influenza viruses possessing seven A/Hong Kong/156/97 (H5N1)–like genes in poultry. J Virol. 2002;76:507–16. 10.1128/JVI.76.2.507-516.2002 11752141 PMC136834

[20] Hoffmann E, Stech J, Leneva I, Krauss S, Scholtissek C, Chin PS, Characterization of the influenza A virus gene pool in avian species in southern China: was H6N1 a derivative or a precursor of H5N1? J Virol. 2000;74:6309–15. 10.1128/JVI.74.14.6309-6315.2000 10864640 PMC112136

[21] Claas EC, Osterhaus AD, van Beek R, De Jong JC, Rimmelzwaan GF, Senne DA, Human influenza A H5N1 virus related to a highly pathogenic avian influenza virus. Lancet. 1998;351:472–7. 10.1016/S0140-6736(97)11212-0 9482438

[22] Yuen KY, Chan PK, Peiris M, Tsang DN, Que TL, Shortridge KF, Clinical features and rapid viral diagnosis of human disease associated with avian influenza A H5N1 virus. Lancet. 1998;351:467–71. 10.1016/S0140-6736(98)01182-9 9482437

[23] Subbarao K, Klimov A, Katz J, Regnery H, Lim W, Hall H, Characterization of an avian influenza A (H5N1) virus isolated from a child with a fatal respiratory illness. Science. 1998;279:393–6. 10.1126/science.279.5349.393 9430591

[24] National Center for Biotechnology Information [database on the Internet]. Influenza virus resource, information, search and analysis. 2008 [cited 2009 Feb 3]. Available from http://www.ncbi.nlm.nih.gov/genomes/FLU/FLU.html

[25] Squires B, Macken C, Garcia-Sastre A, Godbole S, Noronha J, Hunt V, BioHealthBase: informatics support in the elucidation of influenza virus host pathogen interactions and virulence. Nucleic Acids Res. 2008;36(database issue):D497–503. 10.1093/nar/gkm905 PMC2238987 17965094

[26] Paradis E. Analysis of phylogenetics and evolution with R. New York: Springer; 2006.

[27] Felsenstein J. Evolutionary trees from DNA sequences: a maximum likelihood approach. J Mol Evol. 1981;17:368–76. 10.1007/BF01734359 7288891

[28] Drummond AJ, Rambaut A. BEAST: Bayesian evolutionary analysis by sampling trees. BMC Evol Biol. 2007;7:214. 10.1186/1471-2148-7-214 17996036 PMC2247476

[29] Drummond AJ, Rambaut A, Shapiro B, Pybus OG. Bayesian coalescent inference of past population dynamics from molecular sequences. Mol Biol Evol. 2005;22:1185–92. 10.1093/molbev/msi103 15703244

[30] Hasegawa M, Kishino H, Yano T. Dating of the human–ape splitting by a molecular clock of mitochondrial DNA. J Mol Evol. 1985;22:160–74. 10.1007/BF02101694 3934395

[31] R Development Core Team. R: a language and environment for statistical computing. Vienna: R Foundation for Statistical Computing; 2009.

[32] Obenauer JC, Denson J, Mehta PK, Su X, Mukatira S, Finkelstein DB, Large-scale sequence analysis of avian influenza isolates. Science. 2006;311:1576–80. 10.1126/science.1121586 16439620

[33] Beerli P. Comparison of Bayesian and maximum-likelihood inference of population genetic parameters. Bioinformatics. 2006;22:341–5. 10.1093/bioinformatics/bti803 16317072

[34] Beerli P, Felsenstein J. Maximum-likelihood estimation of migration rates and effective population numbers in two populations using a coalescent approach. Genetics. 1999;152:763–73. 10353916 10.1093/genetics/152.2.763 PMC1460627

[35] Beerli P, Felsenstein J. Maximum likelihood estimation of a migration matrix and effective population sizes in n subpopulations by using a coalescent approach. Proc Natl Acad Sci U S A. 2001;98:4563–8. 10.1073/pnas.081068098 11287657 PMC31874

[36] Halvorson D. Control of low pathogenicity avian influenza. In: Swayne DE, editor. Avian influenza. Chichester (UK): Blackwell Publishing; 2008.

