# Supplementary material for: Invasions by Eurasian Avian Influenza Virus H6 Genes and Replacement of Its North American Clade
Source: Emerg Infect Dis. 2009 Jul;15(7):1040–5. doi: 10.3201/eid1507.090245 (PMC2744232; doi:10.3201/eid1507.090245)
Supplement: Technical Appendix — Invasions by Eurasian Avian Influenza Virus H6 Genes and Replacement of Its North American Clade [file 09-0245_Techapp-s1.pdf]

Invasions by Eurasian Avian Influenza Virus H6 Genes and Replacement of Its North American Clade

Technical Appendix

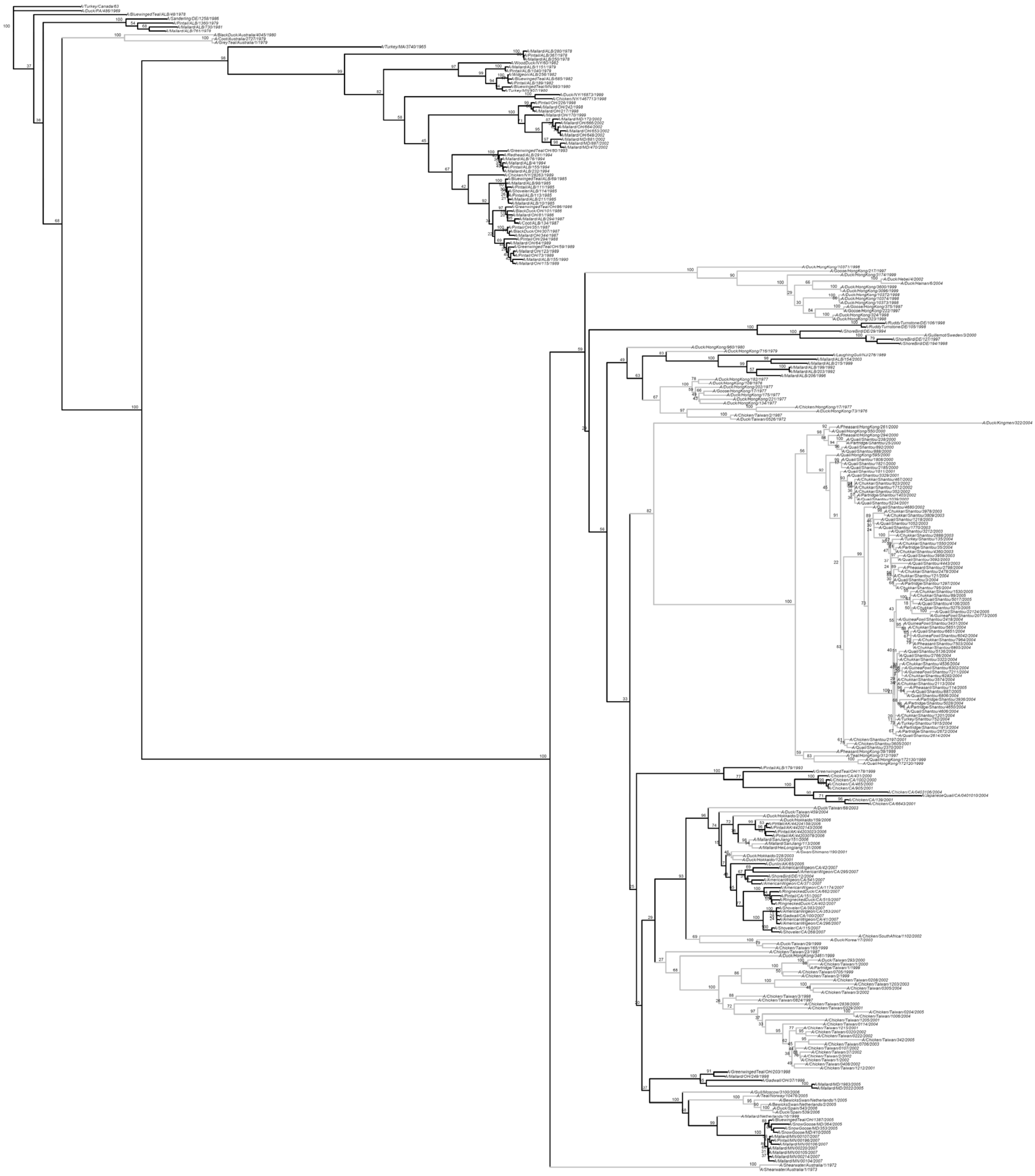

Technical Appendix Figure 1. Complete H6 tree. Gray branches indicate isolates from Eurasia; black branches, isolates from North America.

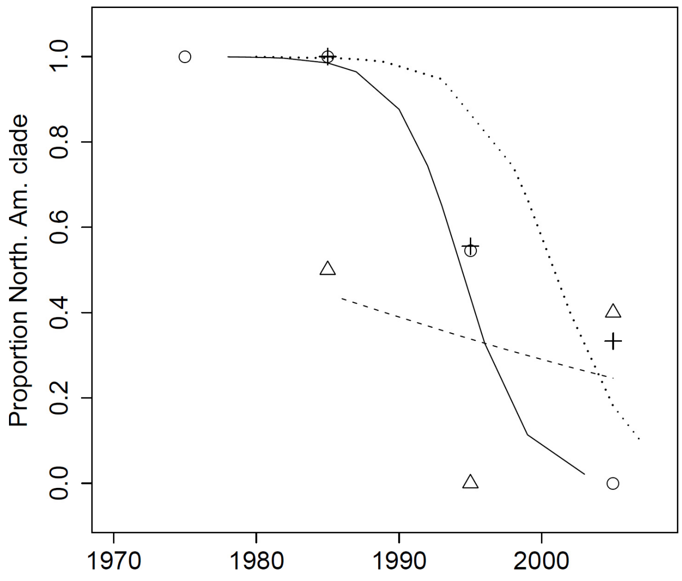

Technical Appendix Figure 2. Proportion of viruses from the North American clade among wild bird isolates in 3 regions of North America grouped by decade. Lines show fitted logistic regressions. Triangles and dashed line show proportions among samples in the Northeast (Delaware and Maryland, p = 0.63, n = 17), crosses and dotted lines show samples from the Midwest (Ohio and Minnesota, p<0.01, n = 34), circles and solid line show samples from Alberta (p<0.05, n = 33).

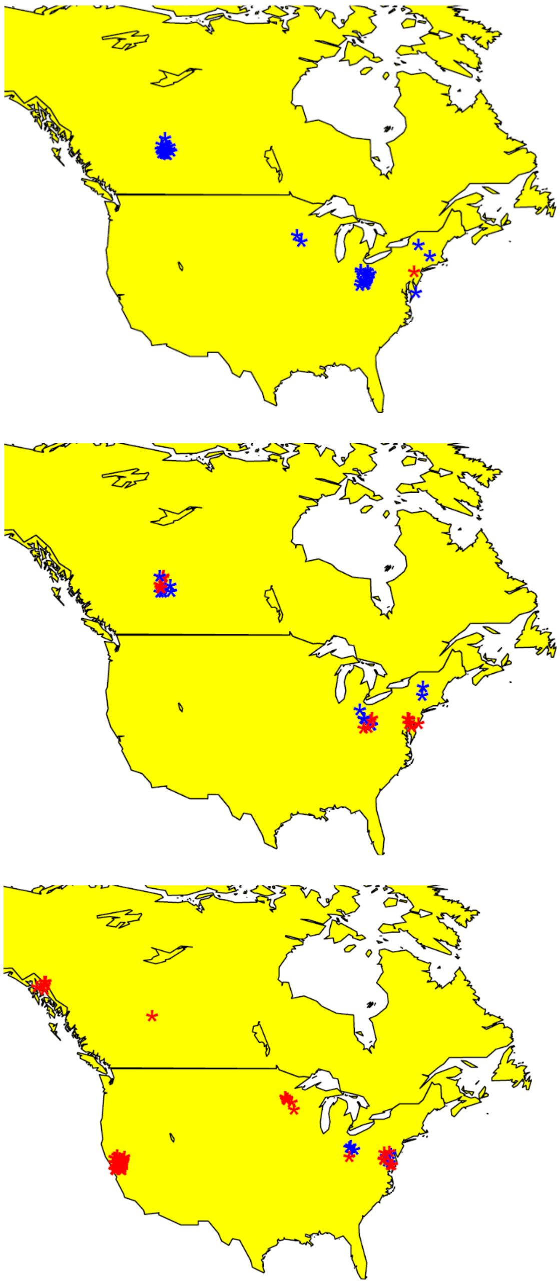

Technical Appendix Figure 3. Maps of H6 distributions in North America at different decades: 1980s (top), 1990s (middle), and 2000s (bottom). H6 from North American clade is shown in blue, from Eurasian clade in red.

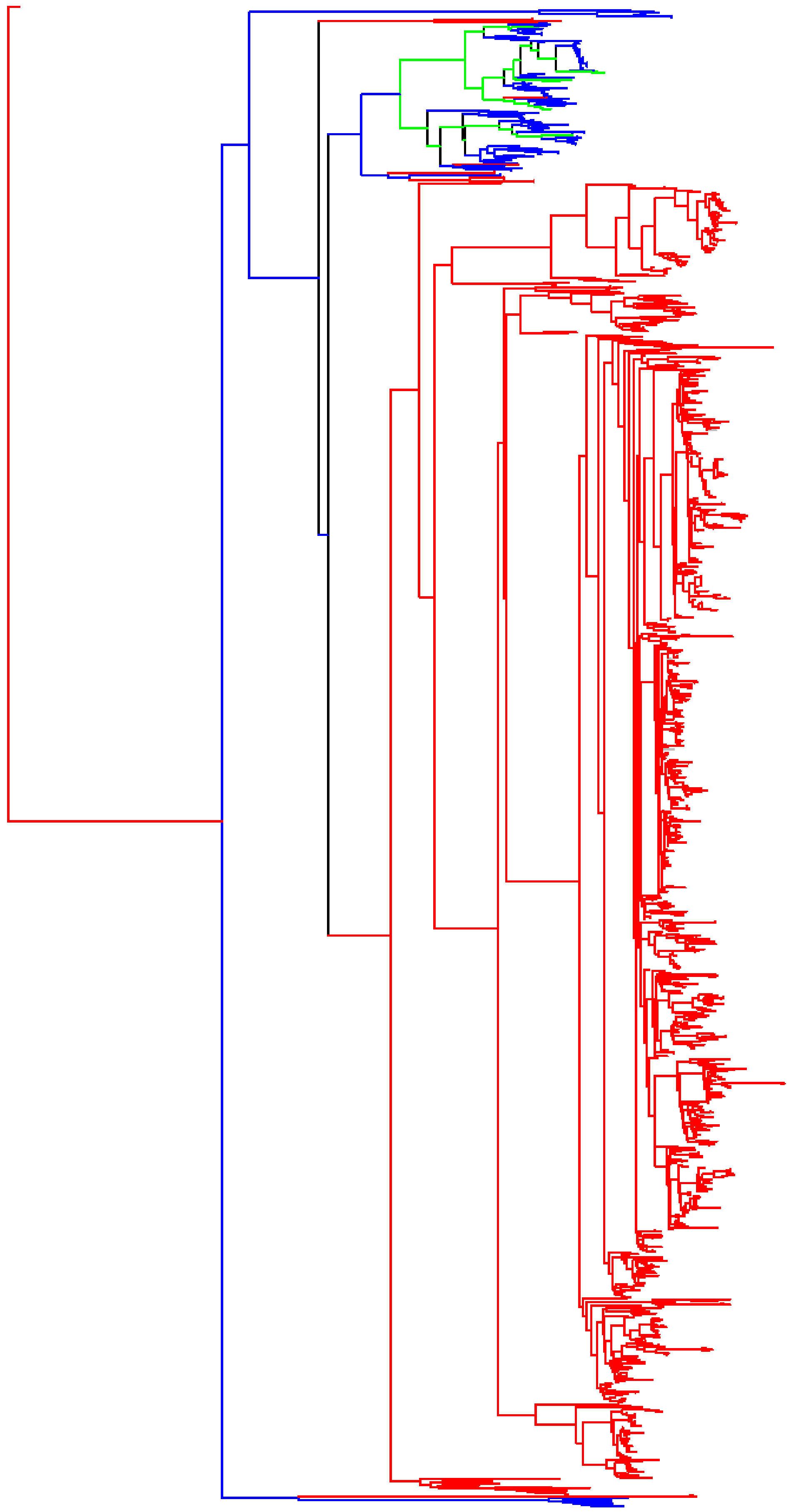

Technical Appendix Figure 4. Complete phylogenetic tree of all N1 nucleotide sequences. The tree was built by the neighbor-joining algorithm. Green branches show isolates whose H6 gene invaded North America from Eurasia, red branches show isolates from Eurasia, and blue branches show isolates from North America. Virus names are not shown because of the large tree size.

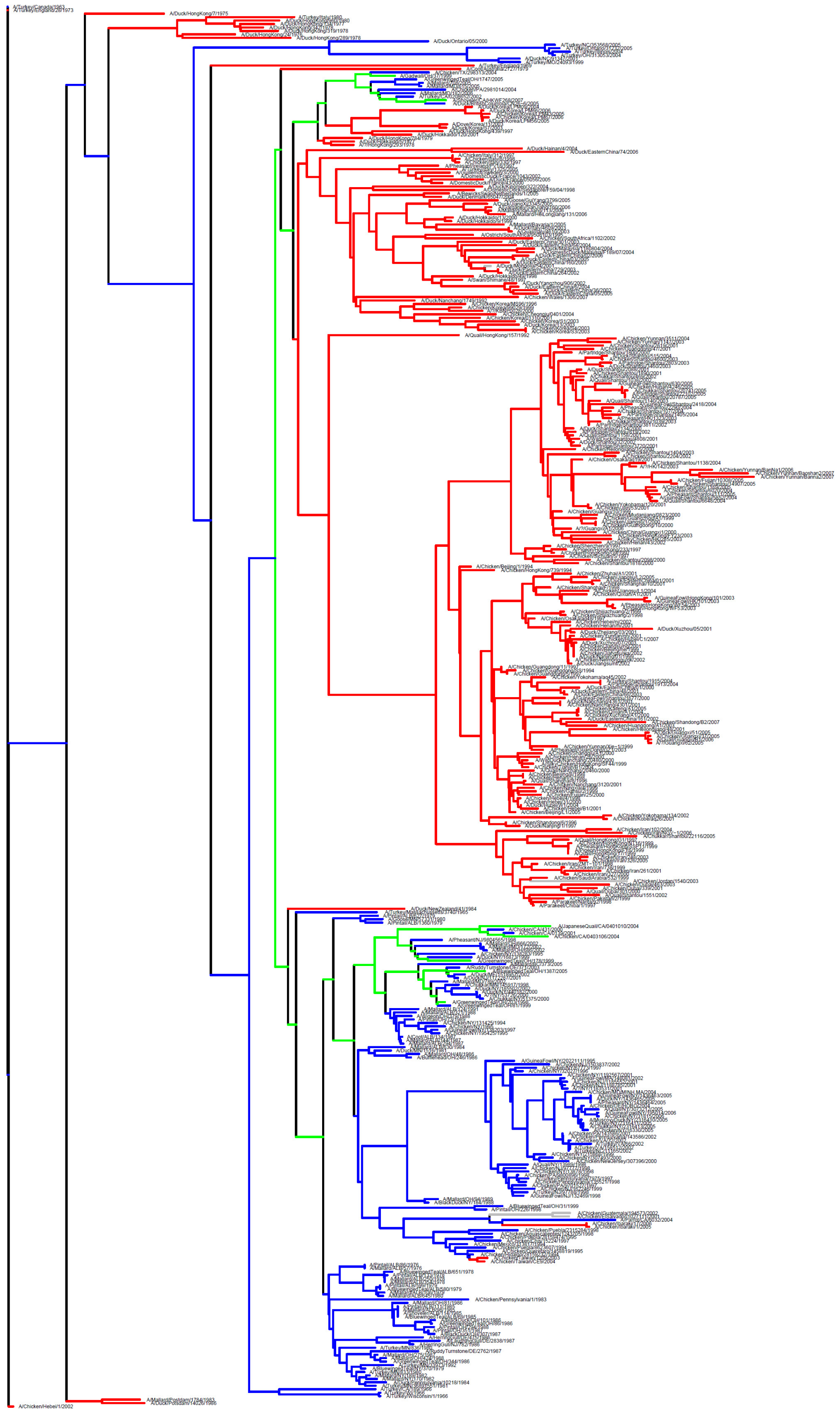

Technical Appendix Figure 5. Complete phylogenetic tree of all N2 nucleotide sequences. The tree was built by the neighbor-joining algorithm. Green branches show isolates whose H6 gene invaded North America from Eurasia, red branches show isolates from Eurasia and blue branches show isolates from North America.

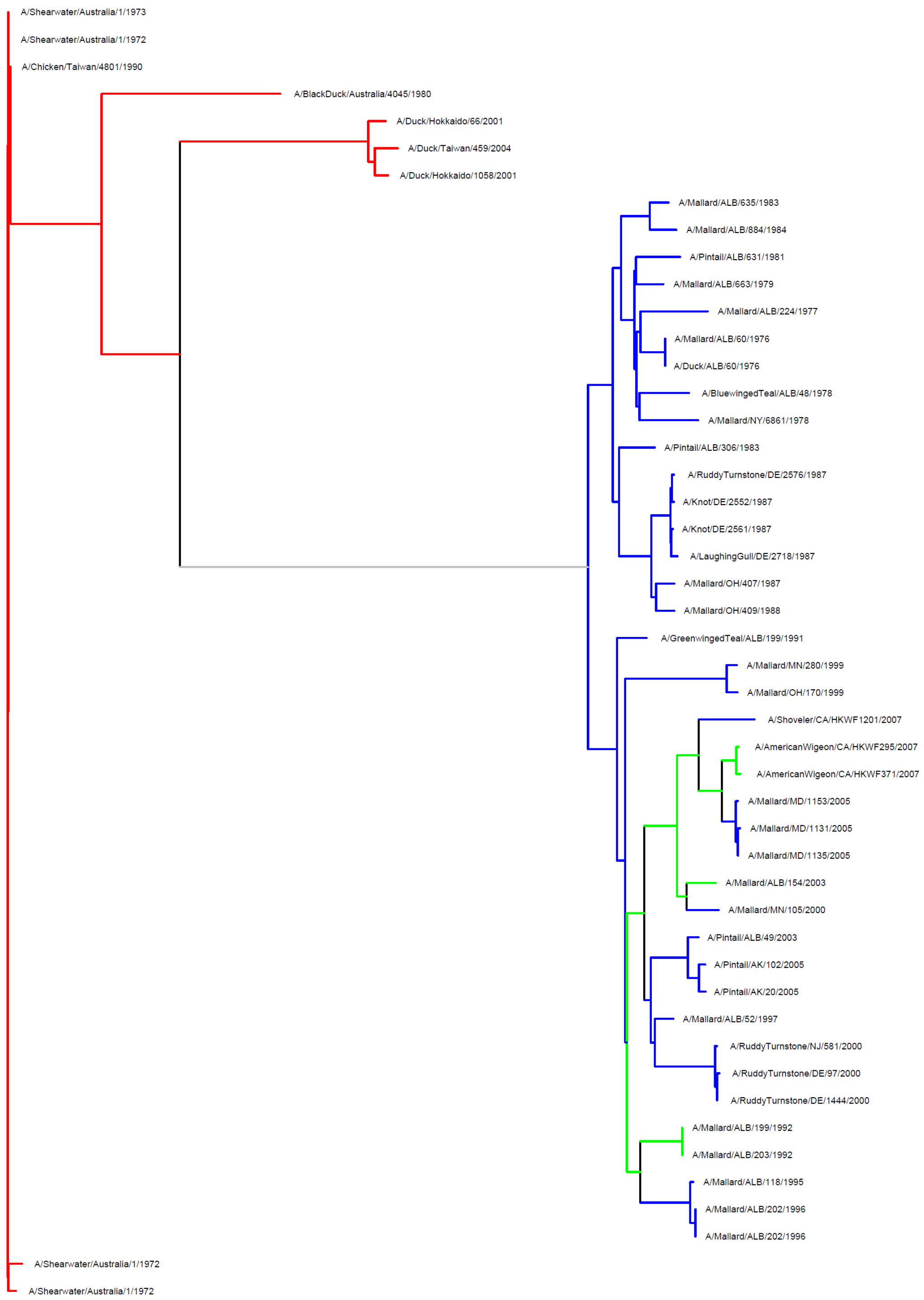

Technical Appendix Figure 6. Complete phylogenetic tree of all N5 nucleotide sequences. The tree was built by the neighbor-joining algorithm. Green branches show isolates whose H6 gene invaded North America from Eurasia, red branches show isolates from Eurasia, and blue branches show isolates from North America.

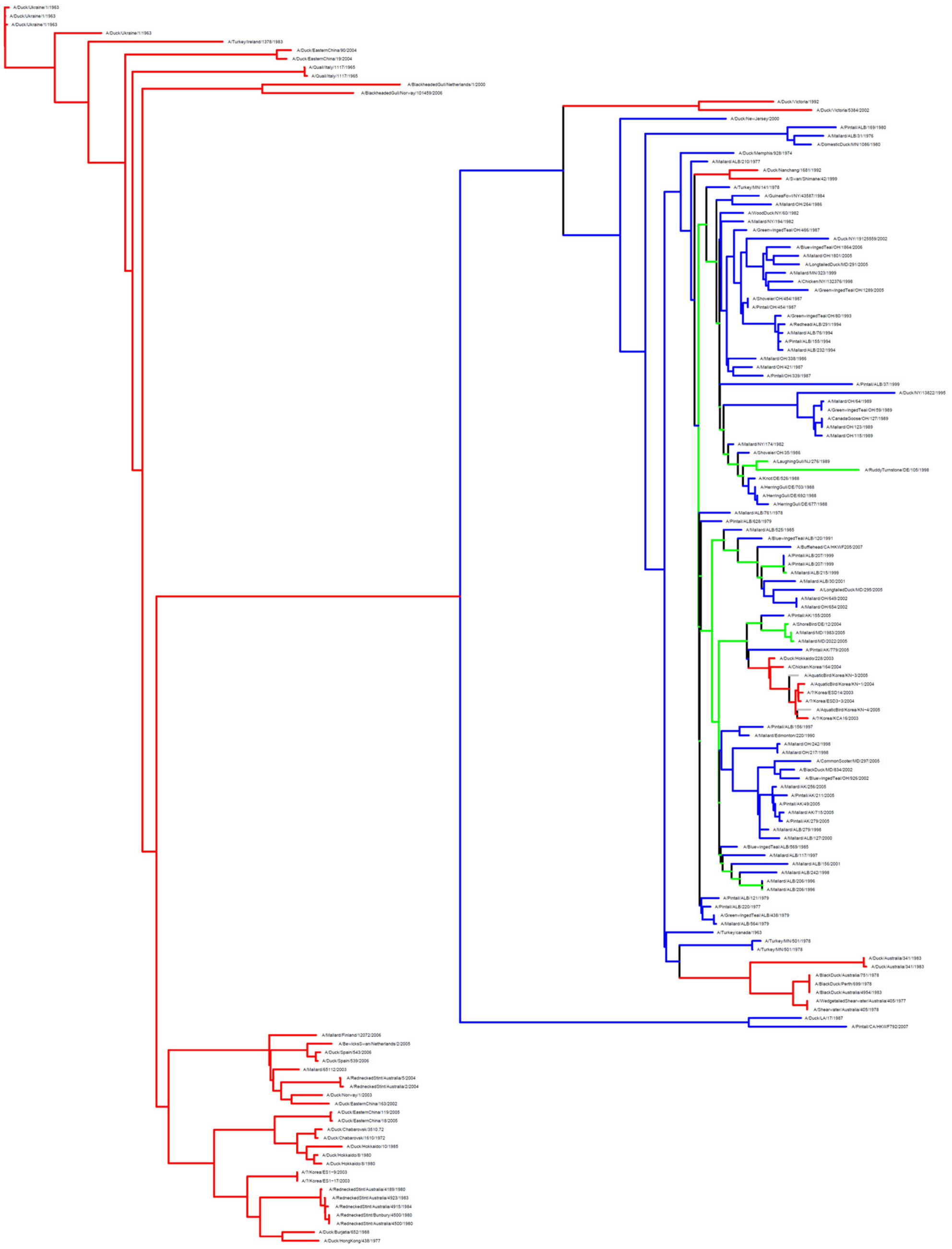

Technical Appendix Figure 7. Complete phylogenetic tree of all N8 nucleotide sequences. The tree was built by the neighbor-joining algorithm. Green branches show isolates whose H6 gene invaded North America from Eurasia, red branches show isolates from Eurasia and blue branches show isolates from North America.

Technical Appendix Table. Abbreviations of bird names and virus sampling locations used in avian influenza virus strain names

Bird full name	Abbreviation	Location full name	Abbreviation
Ruddy turnstone	RT	Delaware	DE
Shorebird	SB	Sweden	SWD
Duck	DK	Hong Kong	HK
Laughing gull	LG	New Jersey	NJ
Mallard	MLD	Alberta	ALB
Pintail duck	PTD	Ohio	OH
Green-winged teal	GWT	California	CA
Chicken	CK	Hokkaido	HOK
Japanese quail	JQL	Alaska	AK
Swan	SW	Shimane	SHM
American widgeon	AWG	Maryland	MD
Ring-necked duck	RND	Minnesota	MN
Dunlin	DL	The Netherlands	NETH
Gadwall	GDW		
Shoveler	SHV		
Blue-winged teal	BWT		
Snow goose	SGS		
Guillemot	GM		
